# The zebrafish cohesin protein Sgo1 is required for cardiac function and eye development

**DOI:** 10.1002/dvdy.468

**Published:** 2022-03-18

**Authors:** Sarah M. Kamel, Sanne Broekman, Federico Tessadori, Erwin van Wijk, Jeroen Bakkers

**Affiliations:** ^1^ Hubrecht Institute‐KNAW, University Medical Centre Utrecht Utrecht The Netherlands; ^2^ Department of Otorhinolaryngology Radboud University Medical Center Nijmegen The Netherlands; ^3^ Donders Institute for Brain, Cognition and Behavior Radboud University Medical Center Nijmegen The Netherlands; ^4^ Department of Medical Physiology, Division of Heart & Lungs University Medical Center Utrecht Utrecht The Netherlands; ^5^ Department of Pediatric Cardiology, Division of Pediatrics University Medical Center Utrecht Utrecht The Netherlands

**Keywords:** cohesinopathy, heart defect, retinal defect, Sgo1, shugoshin, zebrafish

## Abstract

**Background:**

Cohesinopathies is a term that refers to/covers rare genetic diseases caused by mutations in the cohesin complex proteins. The cohesin complex is a multiprotein complex that facilitates different aspects of cell division, gene transcription, DNA damage repair, and chromosome architecture. Shugoshin proteins prevent the cohesin complex from premature dissociation from chromatids during cell division. Patients with a homozygous missense mutation in *SGO1*, which encodes for Shugoshin1, have problems with normal pacing of the heart and gut.

**Results:**

To study the role of shugoshin during embryo development, we mutated the zebrafish *sgo1* gene. Homozygous *sgo1* mutant embryos display various phenotypes related to different organs, including a reduced heart rate accompanied by reduced cardiac function. In addition, *sgo1* mutants are vision‐impaired as a consequence of structurally defective and partially non‐functional photoreceptor cells. Furthermore, the *sgo1* mutants display reduced food intake and early lethality.

**Conclusion:**

We have generated a zebrafish model of Sgo1 that showed its importance during organ development and function.

## Key Findings

- Shugoshin 1 ((SGO1) is part of the cohesion complex and its gene is mutated in patients with CAID syndrome. Here we generated a zebrafish loss‐of‐function mutation for sgo1 and describe that these mutants have defects in cardiac function and eye development leading to early lethality.

## 1 INTRODUCTION

Cohesinopathies is an umbrella term for a group of rare genetic human disorders, which include Cornelia de Lange syndrome (CdLS), Warsaw breakage, Nijmegen breakage, Roberts syndrome, and the most recently identified chronic atrial and intestinal dysrhythmia (CAID).^1^, ^2^ Mutations in genes such as *NIPBL*, *RAD21*, *SMC1A*, *SMC3*, *HDAC8*, and *SGO1* are associated with these diseases and these genes encode for a multisubunit protein complex that mediates sister chromatid cohesion and segregation.^1^, ^2^ This cohesin complex regulates chromatin structure during cell division, gene transcription, and DNA damage repair.^3^, ^4^, ^5^ During cell division, the cohesin complex forms a ring around the sister chromatids to hold them together. Shugoshin 1 (SGO1) works together with protein phosphatase 2A (PP2A) as these proteins act as guardians during chromosome segregation by restricting the removal of the cohesin ring during prophase and to prevent premature sister chromatid separation.^6^, ^7^, ^8^, ^9^, ^10^

CdLS, the most common form of cohesinopathy, is a multisystem disease with a prevalence of 1–3 per 30 000 live births.^8^ It is characterized by developmental delays, mental retardation, craniofacial defects, limb defects, visual and hearing impairment, as well as gastrointestinal and cardiac defects.^2^, ^10^ CAID is a rare form of cohesinopathy that develops due to a homozygous p.(Lys23Glu) mutation in *SGO1*. Patients are asymptomatic at birth, but they start to develop symptoms as early as 6 years of age.^11^ Symptoms of SGO1‐associated CAID patients do not include intellectual disability or growth delays; however, progressive failure in the pace‐making tissue and function, including rhythmic function in the heart and intestinal wall, have been described.^11^

Little is known about the role of SGO1 during development. Mice homozygous for a *Sgo1* loss‐of‐function mutation die in utero indicating that Sgo1 is required during early development. Haploinsufficiency for *Sgo1* results in viable mice, but these display genome instability manifested as mis‐segregation of chromosomes, resulting in enhanced tumorgenesis.^12^ To better understand the role of Sgo1 during development, we generated a zebrafish *sgo1* loss‐of‐function mutant using CRISPR/Cas9 genome‐editing technology. While *sgo1* ^−/−^ larvae develop normally through the first 3 days post‐fertilization (3 dpf), they show a reduced embryonic heart rate, accompanied by reduced end‐diastolic volume, ejection fraction, stroke volume and cardiac output at 5 dpf. Furthermore, eye size of *sgo1* ^*−/−*^ larvae is smaller, retinal lamination is impaired and photoreceptor outer segment morphology is disturbed. The responsiveness to Light ON transitions is also impaired and the larvae have an overall reduced mobility. Due to these defects, early lethality of these mutants progresses within the first 2 weeks of life.

## 2 RESULTS AND DISCUSSION

### 2.1 Generation of *sgo1* mutant zebrafish

**FIGURE 1:**
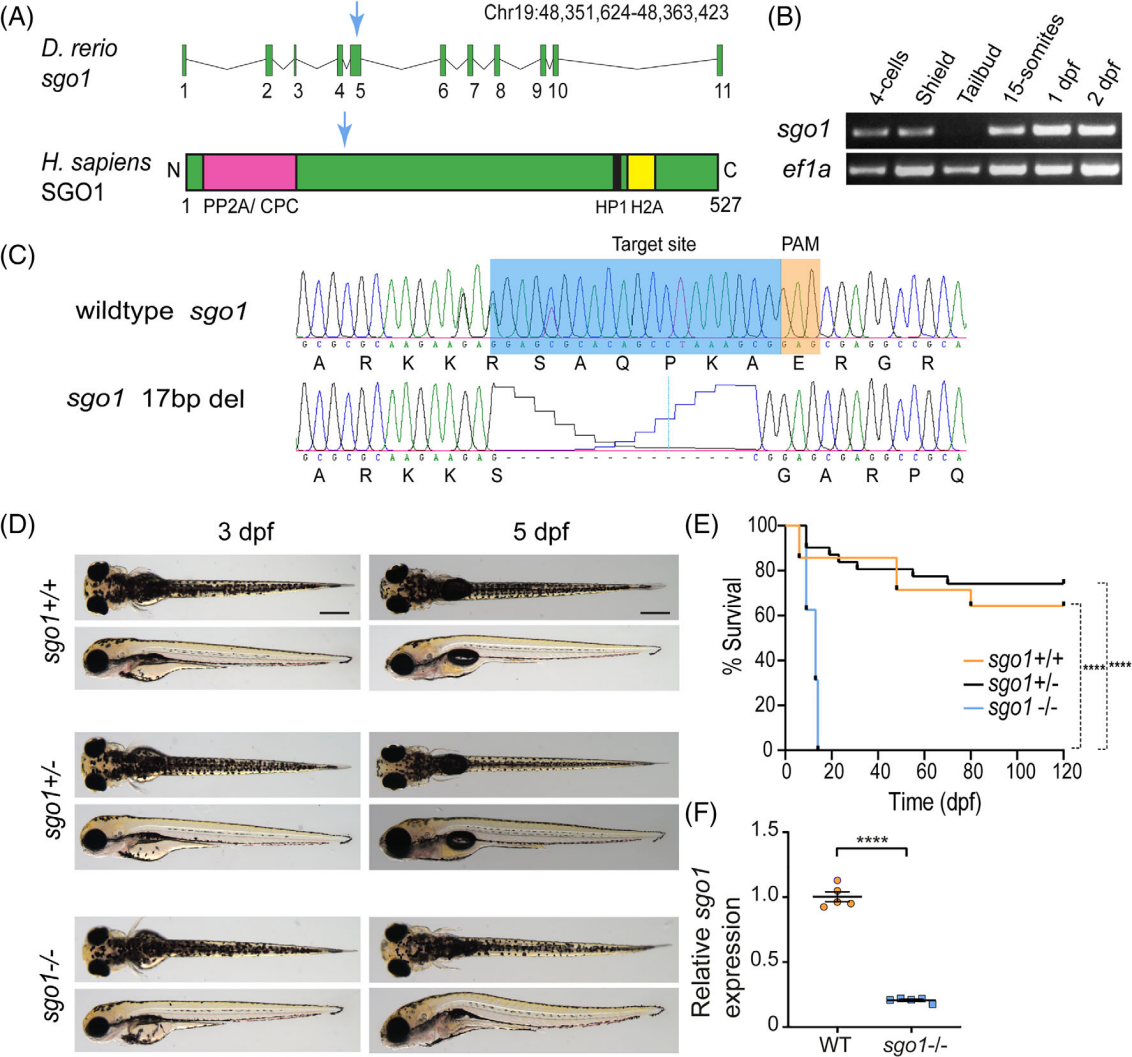
The generation of *sgo1* mutant in zebrafish. (A) *sgo1* transcript in zebrafish (*Danio rerio*) displaying the exons and intron regions. Human (*Homo sapiens*) SGO1 protein, containing 527 amino acids, and binding regions: protein phosphatase 2 (PP2A), chromosome passenger complex (CPC), heterochromatin protein 1 (HP1), and histone H2A (H2A). Blue arrow shows the location of the generated mutant in exon 5. (B) RT‐PCR for *sgo1* and *ef1a* (control) at various developmental stages. (C) CRISPR/Cas9 design for the introduction of a loss‐of‐function mutation in *sgo1*. The mutated sequence shows a 17 bp deletion in exon 5, resulting in an early stop in the mutated sequence and truncation of the protein (not shown). (D) Top and side images of 5 dpf larvae of *sgo1* ^−/−^, *sgo1* ^+/−^ and their wild‐type siblings. (E) Kaplan–Meier curve, displaying the survival of *sgo1* ^−/−^, *sgo1* ^+/−^ and their wild‐type siblings during the first 120 dpf of development (*sgo1* ^+/+^ n = 14, *sgo1* ^+/−^ n = 30, *sgo1* ^−/−^ n = 16). (F) Levels of *sgo1* expression in *sgo1* mutant embryos (n = 5) in comparison with wild type siblings (n = 5). Statistics: (E) Log‐rank (Mantel–Cox) test, mean ± SEM; (F) Two‐tailed nonpaired Student's *t*‐test, mean ± SD; *P* ≤ .0001. Scale bars is at 200 μm. bp: base pairs, PAM: protospacer adjacent motif, dpf: days post fertilization

The zebrafish genome contains a single copy of the *shugoshin‐like 1* gene annotated as *sgo1* (ENSDART00000169729), which is expressed during embryo development (Figure 1A,B). We used CRISPR/Cas9‐mediated genome editing to introduce a loss‐of‐function lesion in exon 5 of *sgo1*. We identified a founder fish containing a 17 bp deletion in exon 5 (Figure 1C) resulting in a frame shift and a premature stop codon leading to the truncation of the predicted protein. A stable line was generated for *sgo1* ^*hu*13334^ and fish were incrossed to generate homozygous mutant embryos for functional characterization (now referred to as *sgo1* ^−/−^). At 3 dpf *sgo1* ^*−/−*^ larvae have a normal size and develop without any visible phenotypes (Figure 1D). At 5 dpf, we observed that *sgo1* ^−/−^ larvae displayed morphological changes such as failure to inflate the swim bladder in 80% of the homozygous mutants and all died within 14 days after fertilization (Figure 1D,E). In addition to the lack of an inflated swim bladder, eye size of *sgo1* ^−/−^ larvae was smaller and the lens angle pointed downward. In about 20% of *sgo1* ^−/−^ larvae, we observed a cardiac edema but heart morphology appeared normal. Furthermore, we observed that *sgo1* mRNA levels were reduced by approximately 80% in *sgo1* ^−/−^ larvae (Figure 1F), likely due to the induction of nonsense‐mediated mRNA decay. From these results, we concluded that Sgo1 has an essential role in embryo development. The absence of earlier phenotypes (before 3 dpf) may be a consequence of maternal *sgo1* mRNA (Figure 1B) and/or protein in the oocyte.

### 2.2 Impaired heart rate and cardiac output in the *sgo1* mutants

**FIGURE 2:**
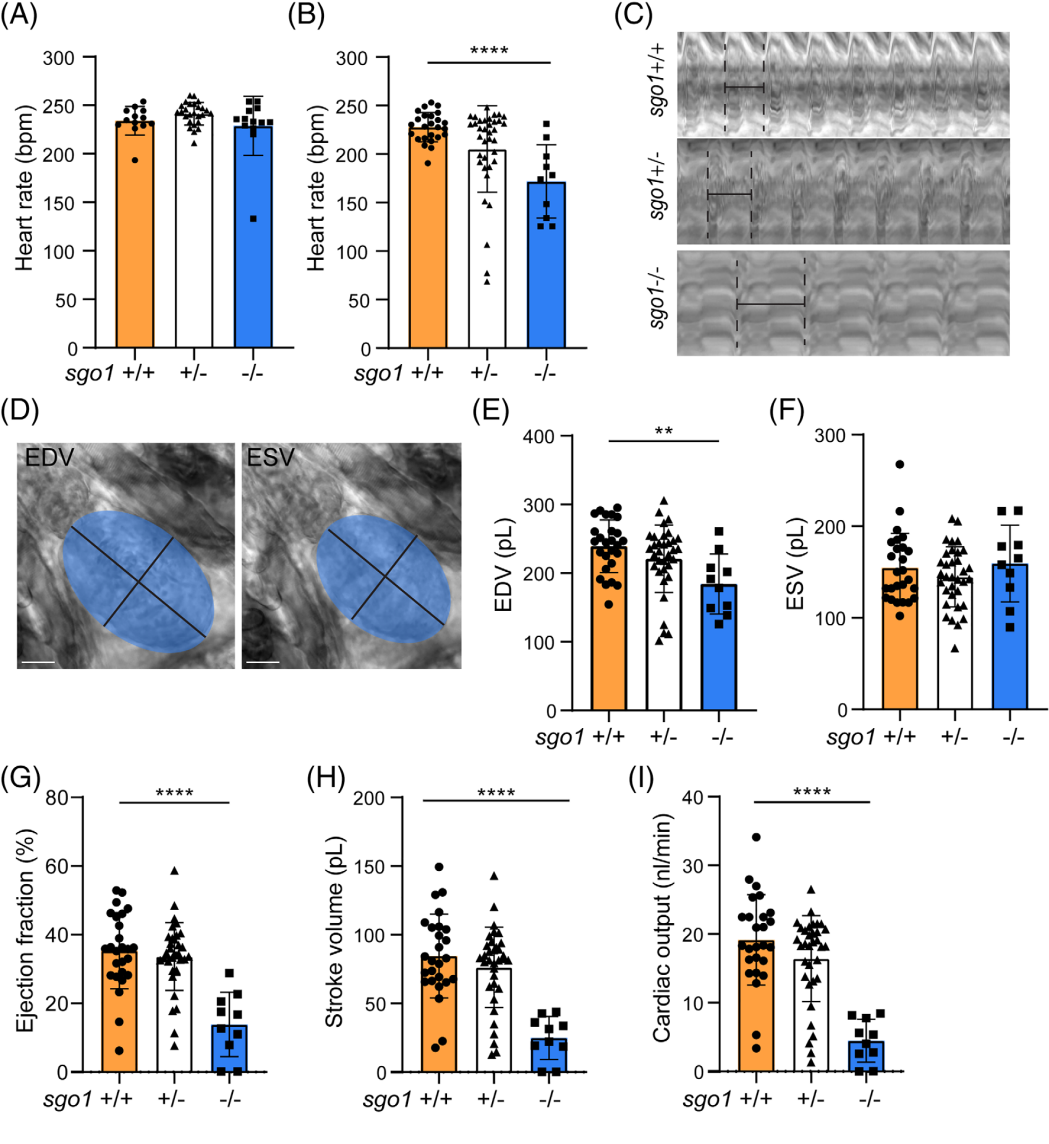
*sgo1* ^−/−^ show functional heart defects at 5 dpf. (A) Heart rate measurements in *sgo1* ^*+/+*^, *sgo1* ^*+/−*,^ and *sgo1* ^*−/−*^ larvae at 3 dpf (*sgo1* ^*+/+*^ n = 13, *sgo1* ^*+/−*^ n = 26, *sgo1* ^*−/−*^ n = 13). (B) Heart rate measurements in wild type, heterozygous carriers and homozygous mutant larvae (5 dpf; *sgo1* ^+/+^ n = 25, *sgo1* ^+/−^ n = 36, *sgo1* ^−/−^ n = 10). (C) Kymograph of beat‐to‐beat distances in wild‐type, heterozygous carriers and homozygous mutant larvae (5 dpf). (D) Sample images of the ellipse taken at end diastolic volume (EDV) and end systolic volume (ESV). Measurements using EDV/ESV ellipse methods for wild‐type, heterozygous carriers and homozygous mutants at 5 dpf include: (E) EDV, (F) ESV, (G) ejection fraction (EF), (H) stroke volume (SV), and (I) cardiac output (CO). All measurements were performed in two biological replicates. Statistics: mean ± SEM, *P* ≤ .05, ***P ≤ .001, ****P ≤ .0001, n.s. *P* > .05, one‐way ANOVA. Bmp: beats per minute, pL: picoliter, nL/min: nanoliter per minute, mm^2^: square millimeter

In our previous work, we showed that *sgo1* is expressed in the zebrafish heart and gut and that a knock‐down of *sgo1* using antisense morpholino oligos resulted in a decrease in heart rate.^2^ We therefore investigated the function of the heart in wild type and *sgo1* ^*−/−*^ larvae using high‐speed video recordings followed by image analysis. At 3 dpf, we were unable to detect any difference in heart rate between wild‐type and *sgo1* ^*−/−*^ larvae (Figure 2A). However, by 5 dpf, there was a significant decrease in heart rate detected in *sgo1* ^*−/−*^ larvae (Figure 2B). Kymograph images of the movies clearly display that *sgo1* ^−/−^ larvae have a longer beat‐to‐beat distance, indicating a slower heart rate, compared to their wild‐type siblings (Figure 2C). Interestingly, *sgo1* ^+/−^ larvae also displayed a significant lower heart rate compared to their wild‐type siblings, albeit this difference was more pronounced in the *sgo1* ^−/−^ larvae. These results corroborated the previously published data derived from *sgo1* morphants.

We next examined the hemodynamic parameters to determine the effect of the mutation on cardiac pump function. The volume of the ventricle was measured by fitting an ellipse shape in the high‐speed imaging recordings of the zebrafish heart at diastole and systole (Figure 2D). Volume parameters were then calculated during diastole and systole using this ellipse and based on the assumption that the cardiac chambers have a spheroid shape. End systolic volume (ESV) were unaffected, but there was a significant decrease in end diastolic volume (EDV) of *sgo1* ^−/−^ compared to wild‐type siblings and *sgo1* ^+/−^ (Figure 2E,F). This end diastolic defect resulted in a significantly reduced ejection (EF) and stroke (SV) in *sgo1* ^−/−^ compared to *sgo1* ^+/−^ and wild‐type siblings (Figure 2G–I). The reduced stroke volume in combination with the reduced heart rate resulted in over 50% reduction in cardiac output (CO) as seen in recorded movies of *sgo1* ^−/−^ compared to *sgo1* ^+/−^ and wild‐type siblings ([Link], [Link]).

Overall, these results demonstrated that *sgo1* ^−/−^ zebrafish mutants develop bradycardia in combination with reduced contractile function by 5 dpf. We previously reported a similar bradycardia phenotype upon knock‐down of *sgo1*.^2^ Cardiac contractility was not measured in this previous report. The observed cardiac phenotype in *sgo1* ^−/−^ larvae correlates well with the expression of *sgo1* in the entire myocardium of the zebrafish heart with most abundant expression in the sinoatrial node, where the pacemaker cells reside.^2^ Corroborating a role for Sgo1 in pacemaker cells is the recent observation that Sgo1 interacts with the potassium/sodium hyperpolarization‐activated cyclic nucleotide‐gated channel 4 (Hcn4) and is required for proper localization of the Hcn4 channel to the plasma membrane.^13^ The Hcn4 channel is required for the so‐called funny current in pacemaker cells and mutations in HCN4 have been identified in patients with sick sinus syndrome and bradycardia.^14^, ^15^, ^16^, ^17^ Whether the observed reduction in contractility in *sgo1* ^*−/−*^ larvae is also due to impaired Hcn4 activity is not clear and needs to be investigated further.

### 2.3 Reduced food uptake in *sgo1* mutant larvae

**FIGURE 3:**
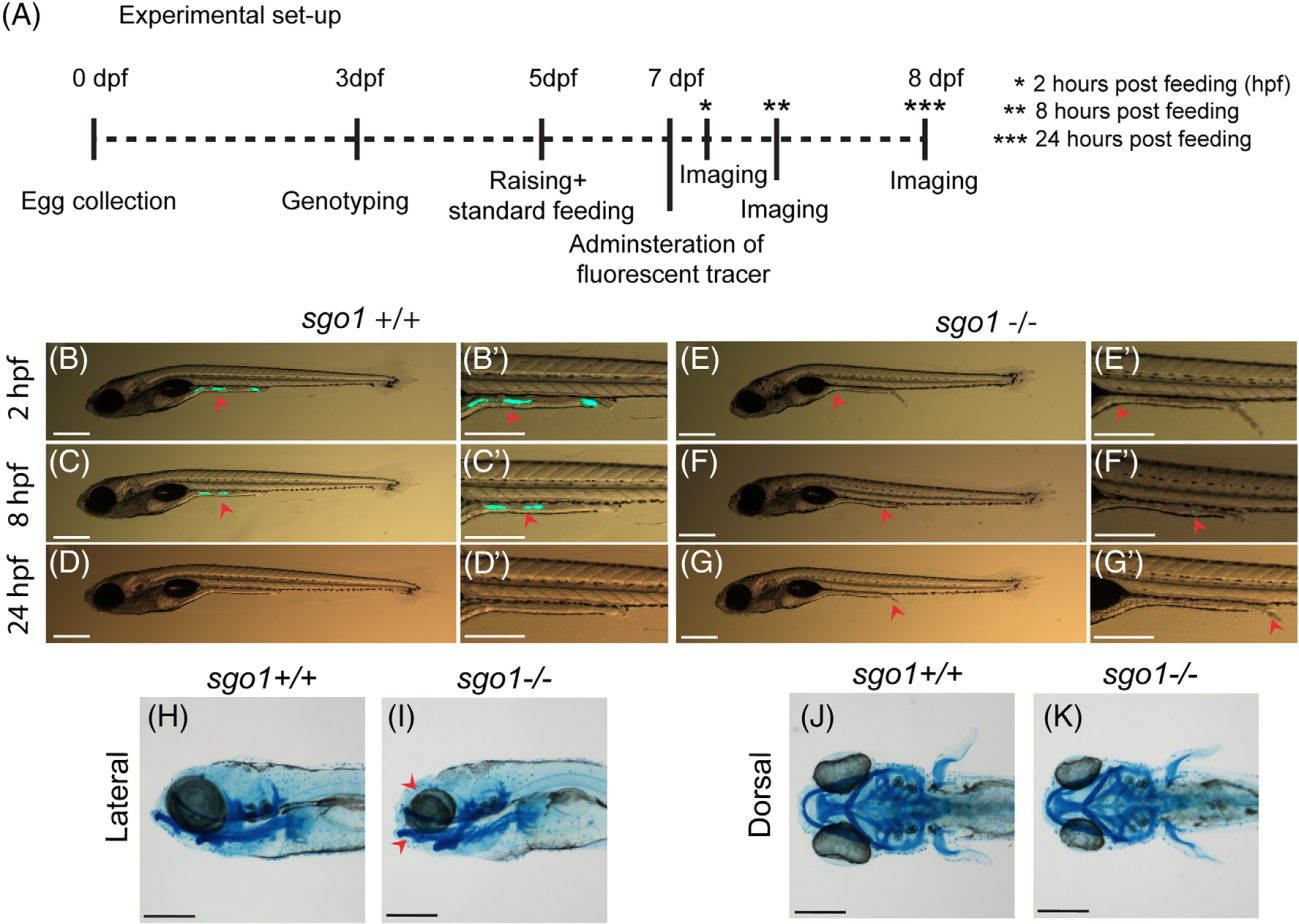
Gastrointestinal defects are not observed in *sgo1* homozygous mutant larvae. (A) A schematic presentation of the intestinal transit assay experimental set up. (B–G) Representative images of 7‐8 dpf larvae at different time points after feeding with fluorescently labelled tracer. Images of the same larvae at different time points are shown. Red arrowheads point to the fluorescently labelled tracer (*sgo1* ^+/+^ n = 10, *sgo1* ^−/−^ n = 10). (H–K) Lateral and dorsal view of Alcian Blue staining in 5 dpf larvae (*sgo1* ^+/+^ n = 20, *sgo1* ^−/−^ n = 21). Scale bars: 200 μm, taken at a magnification of 3.2× for (B–G) and at a magnification of 0.5× for (H–K). dpf: days post fertilization, hpf: hours post feeding

Since *sgo1* is expressed in the developing zebrafish gut and CAID patients display defects in gastrointestinal function,^2^, ^11^ we next examined gut function of *sgo1* mutants. We performed an intestinal transit assay to assess the larval ability to ingest and transit food particles. Larvae derived from a *sgo1* ^*+/−*^ inbreeding were genotyped at 3 dpf. Heterozygous larvae were removed and the remaining larvae were divided into a *sgo1* ^+/+^ and *sgo1* ^−/−^ group. Larvae with a wildtype appearance and visible, fully inflated swim bladder were selected from both groups and raised according to standard conditions until 7 dpf. At 7 dpf, we administered a fluorescently labelled tracer for 2 hours in the morning to each group and then imaged the larval ability to ingest and transit the tracer. Tracing of the fluorescently labelled tracer was performed at 2, 8, and 24 hours post ingestion (Figure 3A). In the wild‐type group, all embryos (n = 10) were able to ingest and transit the tracer within 24 hours of feeding (Figure 3B–D). In the *sgo1* ^−/−^ group, 70% (n = 7) were unable to ingest the tracer (Figure 3E–G). A minority of *sgo1* ^−/−^ larvae (30%; n = 3) were able to ingest the tracer within 24 hours of feeding, albeit at a significantly lower level as compared to their wild‐type siblings (Figure 3G). As jaw defects may hamper food intake, we investigated the presence and morphological appearance of facial skeletal elements in *sgo1* ^*−/−*^ larvae by visualizing cartilage using Alcian Blue. All facial skeletal elements were present and seem to develop normally, except for Meckel's cartilage, which is less protruding compared to their wild‐type siblings (Figure 3H–K). Therefore, we conclude that *sgo1* ^−/−^ larvae have difficulties in their food intake, but once food has been ingested the mutant larvae are able to transport it through their gut.

### 2.4 Retinal development and function are impaired in *sgo1* mutants

**FIGURE 4:**
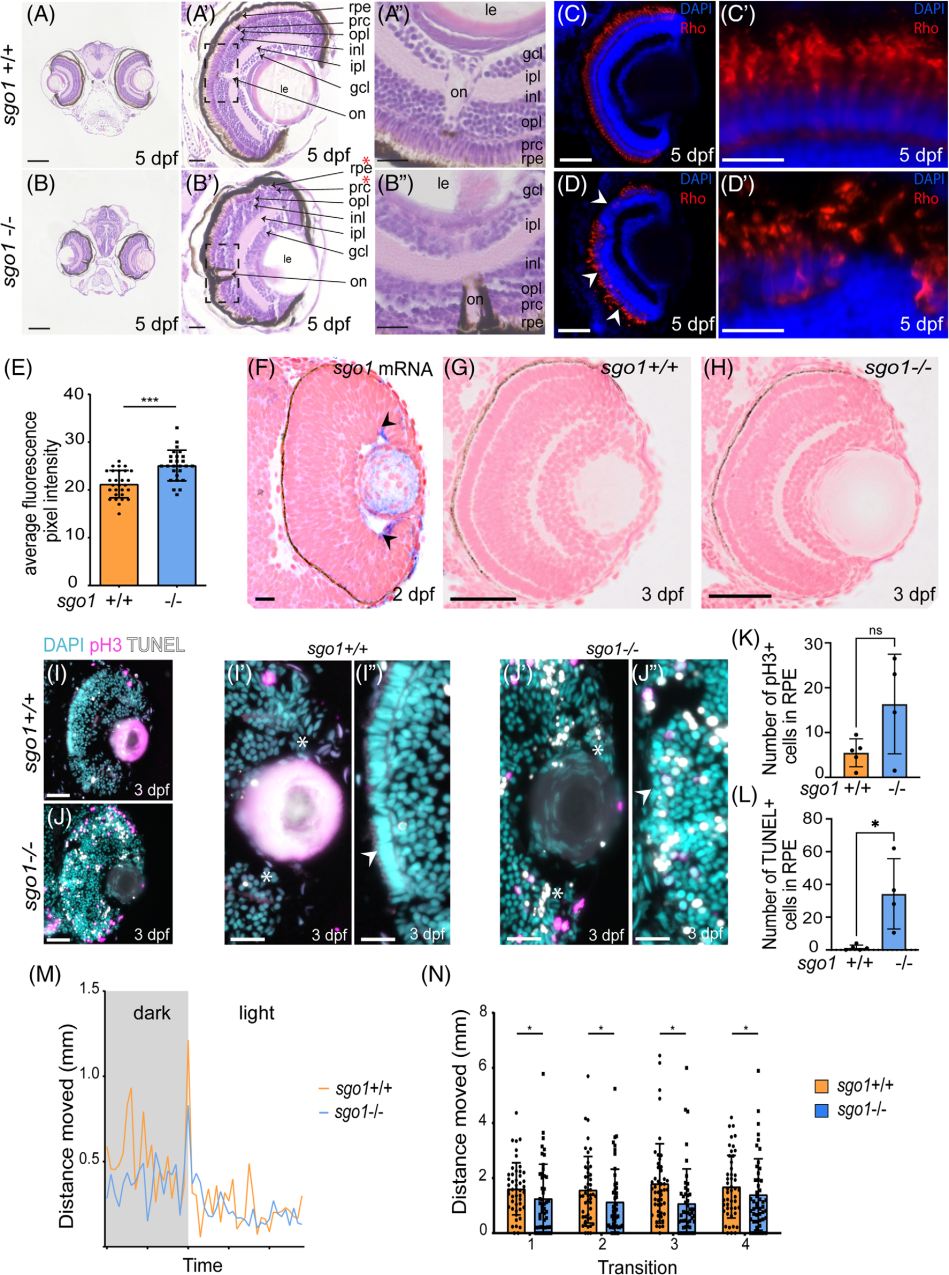
*sgo1* ^−/−^ larvae display morphological defects in the eye, accompanied with slow motility and reduced sensitivity to light/dark cycle. (A, B) Images of wild type and mutants H&E transverse sections of the zebrafish at 5 dpf showing the layers and components of the eye, zoom in of both eyes are shown. rpe: retinal pigmented epithelium, prc: photoreceptor cell layer, opl: outer plexiform layer, inl: inner nuclear layer, ipl: inner plexiform layer, gcl: ganglion cell layer, on: optic nerve and le: lens. Red stars denote the Retinal pigmented epithelia and photoreceptor layer that has been disrupted in the mutants (*sgo1* ^+/+^ n = 6, *sgo1* ^−/−^ n = 6). (C,D) Rhodopsin labeling in retinal cryosections of 5 dpf *sgo1* ^+/+^ and *sgo1* ^−/−^ larvae. (C′,D′) Higher magnifications of retinal regions labeled with Rhodopsin labeling in *sgo1* ^+/+^ and *sgo1* ^−/−^ larvae. (E) Quantification of Rhodopsin localization to the photoreceptor cell bodies of 5 dpf *sgo1* ^+/+^ and *sgo1* ^−/−^ larvae (*sgo1* ^+/+^ n = 24, *sgo1* ^−/−^ n = 24). (F) Transverse section of *sgo1* in situ hybridization on *sgo1* ^+/+^ embryo (2 dpf; n = 6). Black arrows showing the expression in the ciliary marginal zone (cmz). (G,H) Transverse section of *sgo1*+/+ and *sgo1*−/− embryos at 3 dpf. (I,J) Combined mitotic marker (pH3) and TUNEL assay for cell death is shown for 3 dpf *sgo1* ^+/+^ and *sgo1* ^−/−^ larvae (*sgo1* ^+/+^ n = 5, *sgo1* ^−/−^ n = 4). (I′,J") Zoom‐in images of retinal regions in *sgo1* ^+/+^ and *sgo1* ^−/−^ larvae, stem cell niche area is shown with asterisks. (K,L) Quantification of pH 3+ and TUNEL+ cells in RPE of 3 dpf *sgo1* ^+/+^ and *sgo1* ^−/−^ larvae (*sgo1* ^+/+^ n = 5 and *sgo1* ^−/−^ n = 4). (M,N) Visual function recorded by visual motor behavioral assay showing the distance moved by the larvae in both groups during light ON transitions. (M) Average one light ON response recorded in 5 dpf *sgo1* ^*−/−*^ and *sgo1* ^*+/+*^ larva (*sgo1* ^+/+^ n = 48, *sgo1* ^−/−^ n = 48). The grey region represents the period in darkness; the white region represents the period in light. (N) The total distance moved by larvae of both groups during the first second after four consecutive light ON transitions. Functional measurements were performed in two biological replicates. Each data point represents a single larva (*sgo1* ^+/+^ n = 48, *sgo1* ^−/−^ n = 48). Scale bar is at 200 μm for tile scan images taken at a magnification of 20×, and 25 μm for zoom‐in of eye at a magnification of 40× (for A–D). Photoreceptor cell images in C′,D′ were taken at a magnification of 63× with a scale bar of 25 μm. Retinal image in F was taken at a magnification of 63x with a scale bar of 10 μm. Retinal image in G and H were taken at a magnification of 40× with a scale bar of 20 μm. Retinal images in I and J were taken at a magnification of 40× with a scale bar of 50 μm. Retinal images in I′,I″, J', and J" were taken at a magnification of 40× with a with a 200% digital zoom in and scale bar of 10 μm. Statistics: mean ± SEM,***P ≤ .001, n.s. *P* > .05, unpaired Students *t*‐test. S: seconds, mm: millimeter

As the eyes of *sgo1* ^−/−^ larvae were smaller, as well as different in shape and position, we further investigated the retinal structure and visual function of these mutant larvae. H&E staining on retinal sections of 5 dpf larvae revealed that the photoreceptor cell layer and retinal pigmented epithelium (RPE) are disrupted in *sgo1* ^−/−^ embryos (Figure 4A,B, denoted by red asterisks in Figure 4B'). Photoreceptors are sensory neurons that contain light‐detecting specialized cells, they transmit light into signal that is carried to the brain to interpret images.^18^ RPE supports the photoreceptor by maintaining the blood‐retina barrier, transport materials to underlying choroid vessels, secrete factors/signaling molecules that are important for photoreceptor function, and is involved in photoreceptor outer segment disc shedding. Photoreceptor and RPE degeneration and dysfunction lead to vision loss and blindness. Retinal defects have not been observed in patients with CAID;however, we cannot rule out the possibility that they may develop eye defects at later stages of the disease. Interestingly, patients that carry mutations in other cohesin genes such as DDX11 or Rad21 have shown eye defects such as coloboma of the right optic disc and peripheral sclerocornea, respectively.^19^, ^20^

To study photoreceptor function at the molecular level, we investigated the subcellular localization of the photopigment rhodopsin. Rhodopsin is present at the rod outer segments of wildtype larvae as expected (Figure 4C,C'); however, rhodopsin is significantly mislocalized to the photoreceptor cell bodies of *sgo1* ^*−/−*^ larvae (Figure 4D,D',E ). Furthermore, we observed that *sgo1* ^*−/−*^ larvae displayed an irregular morphology of photoreceptor outer segment (Figure 4C',D'), and occasionally regions within the retina with a complete absence of photoreceptor cells.

To investigate how Sgo1 function relates to the observed disruption of the retinal morphology, we performed *in situ* hybridization to detect *sgo1* mRNA expression in 2 dpf wild‐type embryos. We observed a weak but consistent expression of *sgo1* in the ciliary marginal zone (CMZ) (Figure 4F, black arrows), where the retinal stem cell niche is located.^21^, ^22^, ^23^ Mutants at 3 dpf do not display phenotypic changes in the retina, those changes are apparent later at 5 dpf (Figure 4G,H). The retina of the zebrafish undergoes constant renewal throughout life, relying on retinal stem cells (RSCs), which are the youngest and less determined cells at the periphery, and retinal progenitor cells (RPCs) that are the more quiescent and differentiated cells located more centrally in the CMZ.^21^, ^22^, ^23^ To visualize any proliferation defects or apoptotic events, staining with the mitotic marker anti‐phospho Histone H3 (pH3) and TUNEL assay was performed at 3 dpf. In *sgo1* ^−/−^, increased pH3‐ and TUNEL‐positive cells were seen in all layers of layers of the retina and RPE (Figure 4I,J), with the exception to the area of the stem cell niche (Figure 4 I',I'',J',J''). Quantification of the pH3 and TUNEL positive cells in the RPE revealed a significant increase in TUNEL+ cells in *sgo1* ^−/−^ larvae and a trend of more pH3+ cells; however, this difference was not significant (Figure 4K,L). These results indicate that the retinal cells die due to the lack of Sgo1 leading to incomplete growth of the PRC and changes in the structure of RPE. Why retinal cells die in *sgo1* mutants is not clear and needs to be investigated further.

To examine the consequence of these structural differences on the level of visual function, we recorded their response to light using a behavioral visual motor response (VMR) by exposing larvae to alternating light–dark transitions. *sgo1* ^−/−^ larvae show a significantly less pronounced light ON‐response compared to their wild‐type siblings as shown by a significant decrease in distance covered after a dark‐to‐light transition (Figure 4M,N). Altogether, we can conclude that retinal morphology is severely impaired in *sgo1* ^−/−^ larvae resulting in a reduced visual perception and sensitivity to light stimuli.

In this study, we generated a zebrafish *sgo1* loss‐of‐function mutant and studied its role in zebrafish development. We found that *sgo1* ^*−/−*^ larvae at 5 dpf have a significant decrease in heart rate as well as functional cardiac defects resulting in reduced cardiac output. In addition, phenotypical changes in the eye were observed due to disruption in photoreceptor layer and reduced sensitivity to dark/light cycle. Furthermore, the *sgo1* ^*−/−*^ larvae show a reduced ability to ingest food, which may be related to either one of the other phenotypes observed such as malformed jaw, impaired vision, and a general asthenia. Together, this results in lethality of the *sgo1* ^*−/−*^ larvae by 2 weeks after fertilization. The cardiac arrhythmia phenotype reported here for *sgo1* ^−/−^ larvae resembles somewhat the arrhythmias seen in CAID patients with a homozygous p.(Lys23Glu) mutation in *SGO1*. On the contrary, the reduced vision of *sgo1* ^−/−^ fish larvae we report here has not been reported for CAID patients. This could indicate that the missense mutation in SGO1 in CAID patients acts as a hypomorphic mutation while the zebrafish *sgo1* deletion reported here causes a much stronger or complete loss of Sgo1 activity. Indeed, the *sgo1* heterozygous larvae display the cardiac arrhythmia phenotype (Figure 2B), while they lack any of the other phenotypes that we report here for the homozygous *sgo1* mutants. Alternatively, shugoshin1 may have different functions in humans compared to zebrafish, which requires further investigation.

## 3 MATERIALS AND METHODS

### 3.1 Zebrafish husbandry

Fish used in this study were housed under standard conditions as described previously.^24^ All experiments were conducted in accordance with the ethical guidelines and approved by the local ethics committee of the Royal Dutch Academy of Sciences (KNAW).

### 3.2 Generation of mutant lines

The *sgo1* ^hu13334^ allele was generated in the wild‐type Tupfel Longfin (TL) strain zebrafish using CRISPR/Cas9 technology. One‐cell‐stage zebrafish embryos were microinjected with an injection mixture containing of (final concentrations): 150 ng/μL nuclear Cas9 (nCas9) mRNA, 20–40 ng/μL sgRNA and 10% (v/v) Phenol Red. Each putative founder adult fish was crossed with a wild‐type adult fish (F1), F1 fish were out‐crossed to wild type at least two times prior to starting the experiments. Homozygous fish were generated by inbreeding heterozygous mutant carriers. The sequence of the sgRNA is GGGGAGCGCACAGCCTAAAG and genotyping primers are as follows: forward: TGTGTGAATGTGTCTTGACAGG and reverse: AAATCAAAGGTGTCGTTTCCTC. *sgo1* ^hu13334/+^ will be referred to as *sgo1* ^+/−^ in the remainder of the study.

### 3.3 Quantitative RT‐PCR

Embryos at 5 dpf from an adult *sgo1* ^*+/−*^ fish incross were used for total RNA extraction using TRIZOL (Life Technologies/Thermo Fisher Scientific, Bleiswijk, the Netherlands; five embryos per extraction). Prior to RNA extraction, embryos were separated phenotypically as siblings or *sgo1* ^*−/−*^ (phenotype described in the main text). In vitro synthesis of cDNA was carried out with RT Superscript III (Life Technologies/Thermo Fisher Scientific). The obtained cDNA was used as template for qPCR using CFX Connect96 Real‐Time System (Bio‐Rad Laboratories, Hercules, CA) and Luminaris HiGreen qPCR Master Mix (ThermoFisher Scientific). Expression of *sgo1* was assessed using primers 5′‐CAGAAGAAGAGCTTCCAGCAG‐3′ and 5′‐GGCCAGAGCTTTATTGTTGG‐3′, respectively, positioned on exon 1 and exon 2 of *sgo1*. Relative gene expression (2−∆∆Ct) was calculated after normalization of *sgol1* expression to the average expression observed in sibling embryos using *eef1g* as a reference gene (primers used: 5′‐TCGTCTGAAGATTGCGAGTG‐3' and 5′‐ACCCTGGTAAGCTGGAACCT‐3′).

### 3.4 High‐speed brightfield imaging

Embryos were placed in 200 μM of 1‐phenyl‐2‐thiourea (PTU) 20–24 hours post fertilization (hpf) to prevent pigmentation. At 3 and 5 dpf, embryos were embedded in 0.3% agarose prepared in E3 medium containing 16 mg/mL MS‐222. Recordings were performed at 150 frames per seconds (fps) using a high‐speed inverted light microscope at 28°C. Whole larvae were then genotyped. Heart rate measurements and contractility parameters were analyzed using ImageJ (U. S. National Institutes of Health, Bethesda, Maryland, USA). Hemodynamic parameters such as volumes and surface areas were analyzed using ImageJ by drawing an ellipse on top of the ventricle at end‐diastole and end‐systole. Per heart, six ellipses were analyzed: three at diastole and three at systole. Values were averaged. ImageJ provided the values for the minor and major axis of each ellipse. End diastolic and end systolic volume (EDV/ESV) were calculated by: (1/6)*(π)*(major axis)*(minor axis^2^). Stroke volume (SV) by: EDV‐ESV. Ejection fraction (EF) by: SV/EDV. Cardiac output (CO) by: SV*Heart rate.

### 3.5 Intestinal transit assay

Larvae from a *sgo1* ^+/−^ incross were genotyped by tailfin clips at 3 dpf to separate into homozygous mutants and wild types. Healthy‐looking larvae were raised at 5 dpf in two separate tanks according to their genotype and were provided with food (Tetrahymena, NOVO Tom DRY food [JBL] and anthemia mixture) according to the standard Hubrecht fish facility feeding protocol. The fluorescently labelled food was prepared by mixing 100 mg of powdered larvae feed (Royal Caviar, 5–50 μm, BERNAQUA) with 150 μL of fluorescently labelled beads (FluoSpheres Carboxylate‐Modified Microspheres, 2.0 μm, yellow‐green fluorescent (505/515) (Invitrogen) on a watch glass with 50 μL demi water, then spreading the paste on a watch glass in a thin layer to dry in the dark. The fluorescently labelled tracer was then scraped off the watch glass and crushed to a fine powder to store at 4°C in the dark. At day 7, larvae were administered the fluorescent tracer at a concentration of 2 mg per petri dish, in accordance with a published study.^25^ Larvae were fed for 2 hours at the start of the light‐cycle (8:00 to 10:00). Larvae were imaged at 10:00 (T0), then after 6 hours of feeding (T8) and the next day at 8 dpf (T24). Images of the larvae were obtained with a fluorescent microscope (Leica M165 FC) and larvae were kept in the fish facility between timepoints to maintain healthy conditions, while kept without feeding for the period of imaging (24 hours).

### 3.6 Bone and cartilage staining

Larvae at 5 dpf from a *sgo1* ^+/−^ incross were fixed overnight at 4°C in fixation and cartilage staining solution (containing 75% EtOH, Formaldehyde, Acetic acid and Alcian Blue). Fixed larvae were dehydrated in 70%, 95%, and 100% EtOH (10 minutes each), then stored in 4°C until imaging. Before imaging, larvae were genotyped by tailfin clips to separate *sgo1* ^−/−^ from wild‐type siblings. Larvae were washed in 100% MeOH and placed in Murray's solution (Benzyl benzoate: Benzyl alcohol at 2:1) for imaging. Brightfield imaging was performed on a Zeiss Axioplan microscope.

### 3.7 Hematoxylin and eosin staining

Larvae from a *sgo1* ^+/−^ incross were fixed at 5 dpf in 4% paraformaldehyde (dissolved in phosphate buffer containing 4% sucrose) overnight at 4°C. Genotyping was performed by tailfin clips to separate homozygous mutants from wild‐type larvae. Larvae were washed in PBS for 30 minutes at room temperature, then dehydrated in ethanol (30 minutes series at room temperature of 1× 25%, 1× 50%, 1× 70%, 1× 85%, 1× 96%, 3× 100%), washed in butanol at room temperature (3× 30 minutes), kept in paraffin 3× 30 minutes at 58°C before embedding in paraffin. Larvae were sectioned transversely at 6 μm thickness using a microtome (Leica RM2035). Hematoxylin and Eosin (H&E) staining was performed on sections according to standard laboratory protocol for paraffin sections. Images were acquired using a Leica DM4000 B LED upright automated microscope.

### 3.8 In situ hybridization and plastic sectioning

*In situ* hybridization was performed on 2 dpf wild‐type embryos using *sgo1* probe as previously described.^2^, ^26^ Larvae from a *sgo1* ^+/−^ incross were genotyped at 3 dpf by tailfin clips to separate into homozygous mutants and wild types. All embryos were dehydrated in EtOH at 70%, 96%, and 100%, then washed in monomer technoit 8100 (technovit + hardnerI). Embryos in technovit 8100 were embedded on coverslip and left to polymerize overnight at 4°C. Sectioning was performed using Leica 2050 microtome using a triangle glass knife with a mounted waterbath. Sections of 7 μm in thickness were transferred on a noncoated slide and dried overnight at room temperature. Slides were stained for 5 minutes with 0.1% Neutral red (Chroma 1B469 Neutralrot), airdried and cover slipped with Pertex (histolab 00801‐EX). Imaging of stained sections was performed using Leica DM4000 B LED upright automated microscope.

### 3.9 Rhodopsin staining

The rhodopsin staining (1:2000 Rhodopsin 4D2, NBP2‐59690, Novus Biologicals) was performed on pre‐genotyped (by tailfin clips) 5 dpf *sgo1* ^−/−^ and wild‐type larvae cryosections of 7 μm thickness as previously described.^27^, ^28^ Imaging was performed using Zeiss Axio Imager. The amount of Rhodopsin staining in the nuclear layer of the photoreceptors was calculated in ImageJ (U. S. National Institutes of Health, Bethesda, Maryland, USA).

### 3.10 Immunohistochemisty and TUNEL assay

Embryos from a *sgo1* ^+/−^ incross were fixed at 3 dpf in 4% paraformaldehyde and genotyped by tailfin clips to separate homozygous mutant from wild‐type larvae. Three washes of 10 min were performed using 4% sucrose (in phosphate buffer) followed by an 30 minute incubation at room temperature in 30% sucrose (in phosphate buffer). Larvae were embedded in tissue‐freezing medium (Leica, Lot# 03811456), frozen on dry ice, and kept at −80°C. Cryo‐sectioning using Cryostar NK70 (Thermo Scientific, Breda, The Netherlands) was performed to obtain 7 μm thin sections. Staining of *sgo1* ^−/−^ and wild‐type larvae was performed. Using Roche In Situ Cell Detection Kit (TMR red# 12156792910). Sections were rinsed in PBT (PBS +0.1% Tween 20), incubated with collagenase Type II (17101015, Gibco) for 30 minutes at room temperature, then blocked for 1 hour at RT with PBT supplied with DMSO and FBS, incubation with Anti‐phospho Histone H3 (Ser10) at 1:100 (06‐570, Millipore) was performed overnight at 4°C. Slides were washed multiple times in PBT and incubated in labeling solution from the kit, supplied with the secondary antibody Alexa 555 Anti‐rabbit (1:500) (Cat# A‐21428, Invitrogen) and DAPI (1:1000) for 1 hour at RT, washed multiple times in PBT and coverslip was added. Imaging was performed using Olympus Slideview VS200 digital slide scanner.

### 3.11 Visual motor behavior assay

Larvae from a *sgo1* ^+/−^ incross were genotyped by tailfin clips at 3 dpf to separate homozygous mutant from wild‐type larvae. At 5 dpf, larvae were placed separately in a 48 well plate (in 350 μL E3 medium [5 mM NaCl, 0.17 mM KCl, 0.33 mM CaCl_2_, 0.33 mM MgSO_4_ and 1.3 × 10^−5^% methylene blue]) to record their motility and behavior to periods of 50 minutes dark and 10 minutes light using an automated video‐tracking system after 1 hour of acclimatizing in the dark. This method of imaging and video analysis was adjusted from the VMR, as previously described.^29^ Imaging was performed using a DanioVision system and tracked with EthoVision XT14 software from Noldus Information Technology (Wageningen, the Netherlands).

### 3.12 Statistical analysis

Statistical analysis and drawing of graphs and plots were carried out in GraphPad Prism (version 7 for Windows, GraphPad Software). Differences between two groups were analyzed using the unpaired Student's *t*‐test, with the exception of the Kaplan‐Meier curve, where Log‐rank (Mantel‐Cox) test was used. All data are presented as mean ± SEM, and *P* < .05 was considered significant. *P ≤ .05, **P ≤ .01, ***P ≤ .001, ****P ≤ .0001, n.s. *P* > .05. n denotes the number of fish used per dataset.

## AUTHOR CONTRIBUTIONS

**Sarah Kamel:** Conceptualization (equal); data curation (equal); formal analysis (equal); methodology (equal); visualization (equal); writing – original draft (equal). **Sanne Broekman:** Data curation (equal); formal analysis (equal); visualization (equal). **Erwin van Wijk:** Conceptualization (equal); funding acquisition (equal); supervision (equal); writing – review and editing (equal). **Federico Tessadori:** Quantitative RT‐PCR experiment and interpretation of data. **Jeroen Bakkers:** Main funding, study design, data interpretation, supervision, manuscript reviewing and editing.

## Supporting information


**Movie S1** High speed video played in slow motion of *sgo1*+/+ embryo at 5 dpf

**Movie S2** High speed video played in slow motion of *sgo1*+/− embryo at 5 dpf

**Movie S3** High speed video played in slow motion of *sgo1*−/− embryo at 5 dpf
